# Postmenopausal women with osteoporosis and osteoarthritis show different microstructural characteristics of trabecular bone in proximal tibia using high-resolution magnetic resonance imaging at 3 tesla

**DOI:** 10.1186/1471-2474-14-136

**Published:** 2013-04-15

**Authors:** Yun Shen, Yue-Hui Zhang, Lei Shen

**Affiliations:** 1 Department of Orthopaedic Surgery, Xinhua Hospital, Shanghai Jiaotong University School of Medicine, Shanghai, China

## Abstract

**Background:**

Osteoporosis (OP) and osteoarthritis (OA) are two common musculoskeletal disorders that affect the quality of life in aged people. An inverse relationship between OP and OA was proposed four decades ago. However, the difference in microstructure of the trabecular bone of these two disorders by high-resolution MRI (HR-MRI) has not been compared. The primary objective of the study is to explain the actual relationship between OA and OP based on differences between bone microstructure of these two diseases. The secondary objectives are to find out the significance of Euler number and its relationship with other structural parameters, and important role of HR-MRI to reveal the microstructure of trabecular bone directly.

**Methods:**

Totally, 30 women with OP and 30 women with OA (n = 60) were included in this study. Primary OA of hip, knee, as well as spinal arthrosis were diagnosed according to plain X-ray film findings. Osteoporosis was defined based on the latest criteria of World Health Organization (WHO). Structural and textural parameters derived from HR-MRI images of proximal tibia were calculated and compared with special software.

**Results:**

There were significant differences in apparent bone volume fraction, trabecular thickness, mean roundness, Euler number, entropy and inverse different moment between OP and OA patients. In OP group, apparent trabecular separation (Tb.Sp), inertia, absolute value and contrast were positively correlated with Euler number, whereas apparent trabecular number (Tb.N), mean trabecular area, inverse difference and inverse different moment were negatively correlated. Apparent trabecular bone volume fraction (BV/TV), mean trabecular area, mean trabecular perimeter and mean skeleton length negatively correlated with Euler number in OA group. Inverse different moment was the texture parameter, which influenced bone mineral density (BMD) of femoral neck, meanwhile contrast influenced BMD of both great trochanter and Ward’s triangle in OP group. While in OA group, Euler number was the exclusive parameter, which affected BMD of femoral neck and Ward’s triangle.

**Conclusions:**

We found significant differences in microstructure parameters derived from HR-MRI images between postmenopausal women with OP and OA. It convincingly supports the hypothesis that there might be an inverse relationship between OP and OA.

## Background

Osteoporosis (OP) and osteoarthritis (OA) are two common diseases that have negative impact on quality of life of elderly. Osteoporosis represents increased fragility of bone resulting from bone loss and degeneration of bone microstructure, and is regarded as a systemic disease, which can increase the risk of fracture. Osteoarthritis is a kind of disease, which shows degenerative changes to articular cartilage and hyperplasia of bone and other connective tissues. The causes of these two diseases have not been fully understood, as they both are multifactorial diseases and are influenced by genetics, external environment, endocrine system, metabolism, biomechanics and trauma, etc. [1-6]. Although OP and OA are both closely related to the bone, cartilage metabolism and aging process of the body, they are generally considered to be two completely different diseases. Previous studies have found that elderly patients with femoral head resection could maintain good quality of articular cartilage of femoral head, while OA patients could maintain better femoral bone mass [7]. Subsequent studies also found that patients suffering from OA of either hip or knee had higher bone mass in axial bone and peripheral bone than normal people and OP patients [8-10]. In 1970s, scholars proposed that there may exist a negative correlation between OA and OP. Although the relationship between OA and OP remains controversial, this inverse relationship has been more widely accepted [9,11-13]. The relationship may vary according to the location of bone mineral density (BMD) measurements, the type of OA, that is, localized or generalized and the stage of OA [14-17]. An interesting observation shows that both the diseases rarely occur in the same patient, and if OA patients suffer OP fractures, they are usually in elderly than other people, which means OA and relevant factors may have a positive protective effect on OP fractures. However, this subject is still unclear and complicated, and more research needs to be carried out to understand it fully [8,18].

Comparison of BMDs in OA, OP and normal controls showed that BMDs of OA patients were the highest [19]. However, there is sufficient evidence that bone quality is not entirely determined by bone densitometry and that its microarchitecture contributes significantly to bone strength [20-24]. Currently, histomorphometric analysis of bone biopsy remains the clinical gold standard technique for the assessment of trabecular microarchitecture. *In vivo* high-resolution imaging, like high-resolution 3D peripheral quantitative micro-CT (HR-pQCT) [25] or high-resolution magnetic resonance imaging (HR-MRI) [26-33], combined with computer-assisted image analysis is useful to visualize and evaluate the microarchitecture of trabecular bone directly.

High-resolution 3D peripheral quantitative micro-CT may be used limitedly due to high radiation dose, while MRI has the advantage of being non-ionizing and non-invasive, offering multi-planar acquisition. In MRI, the trabecular bone marrow shows high signal intensity, whereas the trabecular bone shows lower signal intensity. However, its in-plane spatial resolution is still restrained by signal-to-noise constraints and is currently similar to the trabecular thickness, which is between 100 and 150 μm. As spatial resolution is similar to the trabecular dimension, partial volume effects arise. Therefore, MRI-derived structural parameters are not identical to true histological dimensions, but highly close in anatomical morphology, considered as apparent value [26,34,35].

In patients with OP, trabeculae have lower strength and are of poorer quality, whereas sclerotic subchondral trabecular bone is found in those with OA. However, the increase of stiffness in OA does not mean higher strength. Ding *et al.*[36] reported that the thickness of trabeculae in early-staged OA patients was found to increase significantly, but the strength of the subchondral trabecular bone was still weaker than healthy controls. Although the relationship between OA and OP has been investigated with regard to subchondral bone plates [37,38] or metabolism properties of bone [39], and we have also found differences in ultrastructural characteristics of trabecular bone of the femoral head by electron microscopy in previous study [40], the *in vivo* microstructure of trabecular bone obtained using HR-MRI has not been compared between these two diseases.

The primary objective of the study is to explain the real relationship between OA and OP populations via differences between bone microstructure of these two diseases. The secondary objectives are to find the significance of Euler number and its relationship with other structural parameters and role of HR-MRI to reveal the microstructure of trabecular bone directly.

## Methods

### Subjects

Sixty postmenopausal women were selected with an average age of 61.85 (49–72) years. To avoid the confounding effect of age and sex, the inclusion criterion was set as postmenopausal women with more than 5 years after menopause. Thirty women were diagnosed with primary OA with obvious radiographic degeneration changes (osteophytes, cystic degeneration and sclerosis of joint space) of the articular surfaces. Besides, they all had pain or dysfunctional complaints of the affected joints. Any patient with osteoporotic findings in X-ray was precluded. Patients with congenital or acquired dysplasia, gout and rheumatoid arthritis were also excluded of OA group. Five patients with OA in hip, 13 with OA in knee, 8 with lumbar spondylosis and 4 with cervical spondylosis were included in the present study. As our patients with OA were distributed in different locations, we could not use a single grading system to evaluate all the patients with the current small sample size. The other 30 patients with OP sustained at least one osteoporotic fracture, diagnosed by dual-energy X-ray absorptiometry (DXA). This included 7 patients with proximal humeral fracture, 11 with distal radial fracture and 12 with vertebral compression fracture. Patients with osteomalacia, multiple myeloma, rheumatoid arthritis and secondary OP due to hormone therapy were excluded from OP group. All patients or their family members provided signed informed consent and the protocol was approved by The Xin-Hua Hospital Ethics Committee, Shanghai Jiaotong University.

### Bone mineral density

Bone mineral density was determined by DXA (Challenger, DMS, Pérols, France). Measurements of the proximal femur were obtained including femoral neck, greater trochanter and Ward’s triangle. The left femur was used for densitometry of the hip because this was used for HR-MRI of the tibia.

### Imaging

Magnetic resonance images were acquired with 3-T Signa systems (General Electric, Milwaukee, WI, USA) and a four-element phased array coil (Nova Medical, Wilmington, MA, USA).

The patient was placed in the scanner in supine position; feet first, the knee in the homogeneous center of the field and head outside the inner bore of the MR scanner. A fast gradient echo sequence was first acquired to localize the image (TR = 23.1 ms, TE = 1.6 ms, flip angle = 65°, FOV = 28 cm, bandwidth = ±62.5 kHz, slice thickness = 5 mm, matrix = 256 × 128, NEX 1.00). Then, a phase-cycled fast imaging employing steady-state acquisition sequence was applied to obtain high-resolution axial images of the proximal tibia (TR = 12.3 ms, TE = 4.4 ms, flip angle = 60°, FOV = 18 cm, Bandwidth = ±97.6 kHz, slice thickness = 1 mm, matrix = 512 × 384, NEX 2.00). The spatial resolution was 0.35 × 0.35 × 1 mm^3^ and the whole scan time was about 11 min.

Images were analyzed using custom software created by following the procedure outlined by Majumdar *et al.*[31]. In short, all images were transferred to a Ximagetools Workstation (Biomechanic Lab, Medical Engineering Institute, Tongji University, Shanghai), which was designed to perform the analysis and run in an UNIX/Linux (debian) operation system.

**Figure 1:**
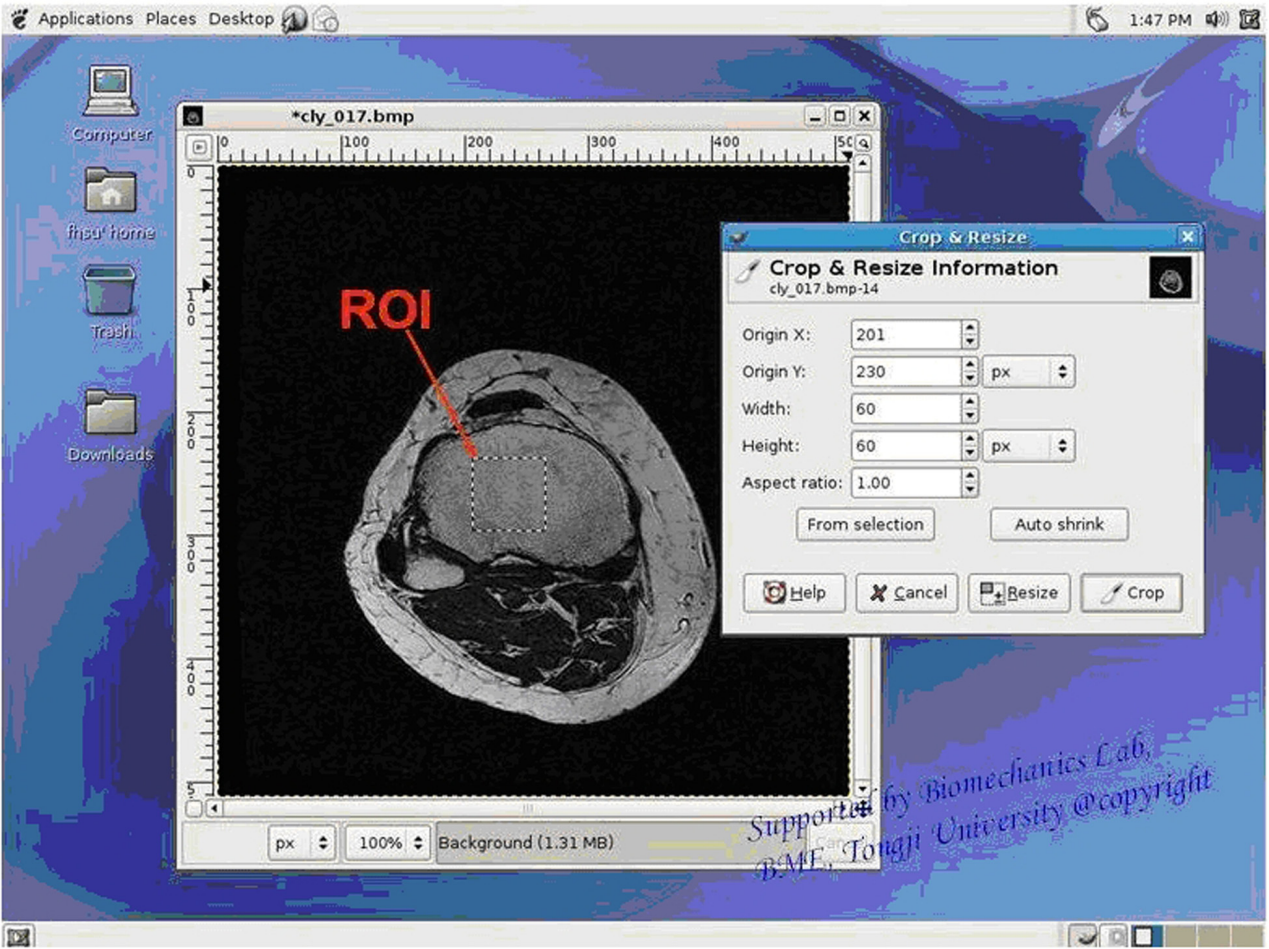
Regions of interest (ROI) containing trabecular bone and marrow in the center of axial plane of the bone.

Prior to the quantitative analysis of the trabecular bone structure, Laplacian of Gaussian filter-based correction algorithm was applied. Image collection began from the metaphysis of the proximal tibia, immediately beneath the subchondral bone, and continued towards the shaft of the bone. The first and last eight images from each set of 36 images were eliminated from the analysis due to different radio frequencies. Moreover, the beginning of image collection varied slightly from patient to patient. Despite the subject-to-subject variation, the mean number of slices analyzed in each bone and in each group was consistent (20 images/bone). The mean value of the structural parameters was calculated from 20 images of each patient. Regions of interest (ROI) containing trabecular bone and marrow in the center of axial plane of the bone were segmented (based on the axial images) at the center of tibia in every image (see Figure 1). Each ROI was square in shape (21 × 21 mm) with 60 × 60 pixels in resolution.

In order to quantify the trabecular bone network, images were, firstly, segmented into a binarized image which contained bone phases in white and marrow phases in dark. During the analysis, the mean signal intensity of the entire ROI was obtained, and a histogram of the signal intensity distribution was plotted. The histogram of signal intensities of bone and marrow included a single peak and an asymmetric tail due to partial volume effect. A global threshold was calculated and the images were binarized into a bone phase and a marrow phase, as previously reported [31].

**Figure 2:**
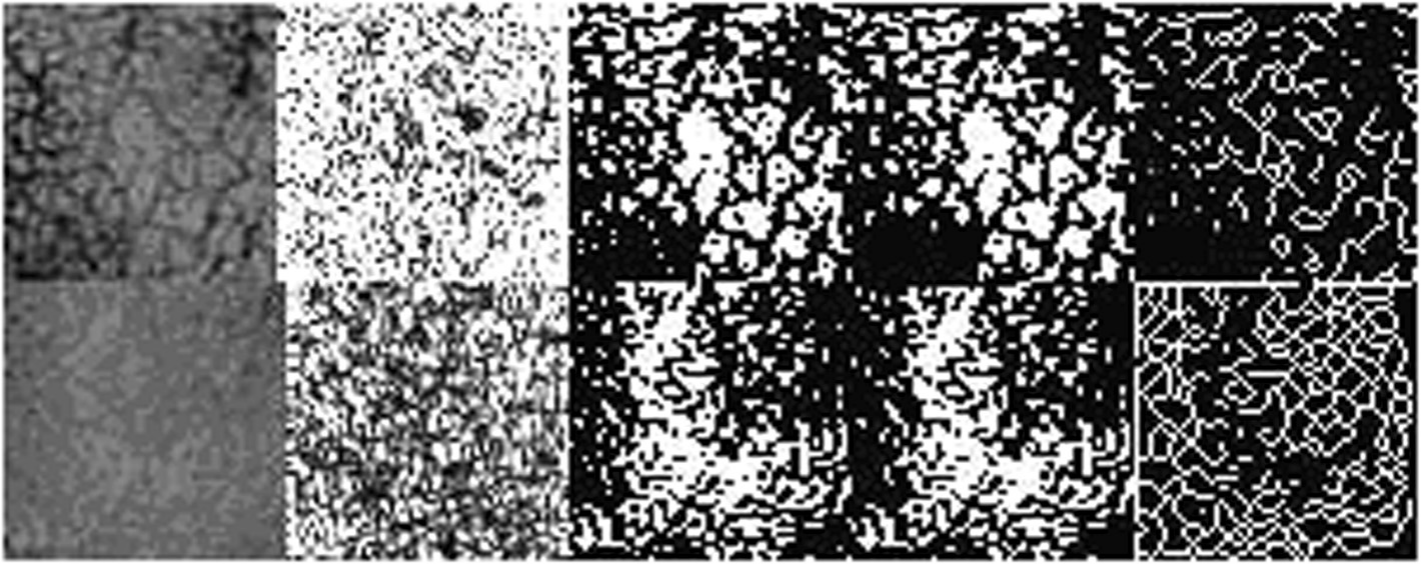
Trabeculae thread.

All the segmented binarized images were scanned for pieces of trabecular bone by Ximagetools, which could divide one image into tens of single trabeculae fragment. Every piece of trabeculae was threaded and number of the pixel of the skeleton was calculated (see Figure 2).

### Outcomes

A total of 15 parameters were derived into three categories as follows:

### Primary outcomes

Structural and topological parameters were studied as primary outcomes.

#### Structural parameters

Structural parameters included the apparent trabecular bone volume fraction (BV/TV), apparent trabecular number (Tb.N), apparent trabecular separation (Tb.Sp), apparent trabecular thickness (Tb.Th), mean trabecular area, mean trabecular perimeter, mean trabecular roundness and mean skeleton length [41].

The total number of pixels contributing to the bone phase relative to the total number of binarized pixels was used to compute app BV/TV; app Tb.Th was computed as the ratio between the total areas of bone pixels relative to the skeleton length; app Tb.N and app Tb.Sp were calculated using app BV/TV and app Tb.Th [42].

Mean trabecular area, mean trabecular perimeter and mean skeleton length were calculated by total trabecular area, perimeter and skeleton length in ROI relative to trabecular number, respectively. Trabecular roundness (R) was computed by the formula (R = 4π*S/C^2^, S represented area, C represented perimeter). The mean value of roundness was taken into the research.

#### Topological parameter

Euler number is a common index reflecting the connection degree of the complex trabecular bone network microstructure. If the value of Euler number is higher, it indicates poorer trabecular bone network connectivity. Euler number and other parameters, which are correlated with both bone quality and strength, have remarkable relevance. Euler number (E), the only topological parameter, was calculated by the difference in the number of connective trabeculae (n) and sealed bone cavity (m) in one unit area.

### Secondary outcome

Texture parameter was studied as a secondary outcome.

#### Texture parameter

Texture parameter included energy, entropy, inertia, absolute value, inverse difference, contrast, angular second moment and inverse different moment. The texture of binarized MRI images reflected bone network directly. We applied spatial gray-level dependence matrix and gray-level difference matrix [43] to analyze the images (Additional file 1).

### Statistical analysis

All values were expressed as mean ± SD. A Mann–Whitney *U* test was used to compare the structural and textural parameters between OA and OP specimens.

To determine the potential association between Euler number and microstructural parameters, including BV/TV, Tb.N, Tb.Th and Tb.Sp, textural parameters, including energy, entropy, inertia, absolute value, inverse difference, contrast, angular second moment and inverse different moment, bivariate correlation analysis was used first to calculate correlation coefficient by using Pearson’s method. To avoid mixed effect from other parameters, partial correlation analysis was performed to find the structural or textural parameter, which related to Euler number more closely.

Multiple linear regression analysis was used to evaluate as to which structural or textural parameter was the most predictable index to influence BMD. Bone mineral density of femoral neck, great trochanter and Ward’s triangle was selected as the dependent variable separately. A stepwise method was used to calculate the value of r^2^.

An SPSS 10.0 software package (SPSS Inc., Chicago, IL, USA) was used for all statistical procedures with the p value of <0.05 being considered significant.

## Results

The Mann–Whitney *U* test demonstrated that there is no significant difference between the age and the menopause time in OA and OP groups (p > 0.05).

All structural parameters, topology parameters and texture parameters calculated by HR-MRI images are listed in Table 1. The Mann–Whitney *U* test demonstrated that the OP and OA patients had significant statistical difference in apparent bone volume fraction, trabecular thickness, mean roundness, Euler number, entropy and inverse different moment (p < 0.05). Inertia and contrast displayed a significant statistical difference even at 1% level of significance (p < 0.01). In OA group, the apparent bone volume fraction, trabecular thickness, roundness and inverse different moment were significantly higher than OP group (p < 0.05). On the contrary, in OP group, Euler number, entropy, inertia and contrast were significantly higher than OA group (p < 0.05).

**Table 1:** Comparison of structural and textural parameters between OP and OA

	**OP (n = 30)**	**OA (n = 30)**	***P***
App BV/TV (%)	0.3471 ± 0.0099	0.3528 ± 0.0121	0.048 ^a^
App Tb.N (mm^-1^)	1.2820 ± 0.1181	1.2566 ± 0.1038	0.381
App Tb.Th (mm)	0.2747 ± 0.0223	0.2856 ± 0.0175	0.041 ^a^
App Tb.Sp (mm)	0.5193 ± 0.0531	0.5275 ± 0.0519	0.547
Mean trabecular area (mm^2^)	2.0808 ± 0.1909	2.1324 ± 0.3576	0.489
Mean trabecular perimeter (mm^2^)	6.9339 ± 0.3294	6.9005 ± 0.4830	0.756
Mean roundness	0.3907 ± 0.0089	0.3953 ± 0.0080	0.043 ^a^
Mean skeleton length (mm^2^)	3.8406 ± 0.2346	3.8124 ± 0.3363	0.708
Total skeleton length (mm^2^)	286.85 ± 18.96	283.55 ± 18.65	0.500
Euler	0.1503 ± 0.0164	0.1397 ± 0.0213	0.035 ^a^
Entropy	1.0578 ± 0.1495	0.95555 ± 0.1975	0.027 ^a^
Energy	0.1403 ± 0.0490	0.1643 ± 0.0660	0.115
Inertia	0.8888 ± 0.2354	0.6983 ± 0.2763	0.006^b^
Absolute Value	0.6284 ± 0.1090	0.5685 ± 0.1359	0.064
Inverse Difference	0.7035 ± 0.0450	0.7319 ± 0.0755	0.082
Contrast	5748.84 ± 2345.99	4144.20 ± 2227.52	0.009 ^b^
Angular Second Moment (×10^-2^)	2.321 ± 0.4913	2.577 ± 0.6877	0.103
Inverse Different Moment	2.1602 ± 1.0454	2.8383 ± 1.1487	0.020^a^

^a^*P* < 0.05,^b^*P* < 0.01.

There was no significant difference between the two groups in apparent trabecular number, mean trabecular area, mean trabecular perimeter, mean skeleton length, total skeleton length, energy, absolute value, inverse difference, angular second moment and other parameters (p > 0.05).

Bivariate correlation analysis showed that in OP group, app Tb.N (r=−0.478), app Tb.Sp (r = 0.503), mean trabecular area (r=−0.918), inertia (r = 0.600), absolute value (r = 0.595), inverse difference (r=−0.587), contrast (r = 0.542), inverse different moment (r=−0.585) were associated with Euler number. Partial correlation analysis showed that after the control of the eight indicators, respectively, only the average trabecular bone area was significantly associated with Euler number (r=−0.625). In OA group, bivariate correlation analysis showed App BV/TV (r=−0.559), mean trabecular area (r=−0.940), mean trabecular perimeter (r=−0.574), mean skeleton length (r=−0.529) were associated with Euler number. However, partial correlation analysis showed that when these four indicators were controlled, the correlation lost statistical significance (see Table 2).

**Table 2:** Correlation analysis between Euler number and structure and texture parameters

	**OP**		**OA**	
	***r***	***P *Value**	***r***	***P *Value**
App BV/TV	−0.269	0.252	−0.559	0.005^b^
App Tb.N	−0.478	0.033^a^	0.039	0.857
App Tb.Th	0.303	0.195	−0.289	0.172
App Tb.Sp	0.503	0.024^a^	0.054	0.803
Mean trabecular area	−0.918	0.001^b^	−0.940	0.001^b^
Mean trabecular perimeter	−0.100	0.676	−0.574	0.003 ^b^
Mean roundness	−0.109	0.647	−0.099	0.646
Mean skeleton length	−0.247	0.294	−0.529	0.008^b^
Entropy	−0.018	0.939	−0.139	0.516
Energy	0.046	0.846	0.164	0.443
Inertia	0.600	0.005b	0.003	0.989
Absolute Value	0.595	0.006^b^	−0.026	0.904
Inverse Difference	−0.587	0.007^b^	0.035	0.871
Contrast	0.542	0.013^a^	−0.348	0.095
Angular Second Moment	0.250	0.288	0.216	0.310
Inverse Different Moment	−0.585	0.007^b^	0.394	0.056

^a^*P* < 0.05,^b^*P* < 0.01.

**Figure 3:**
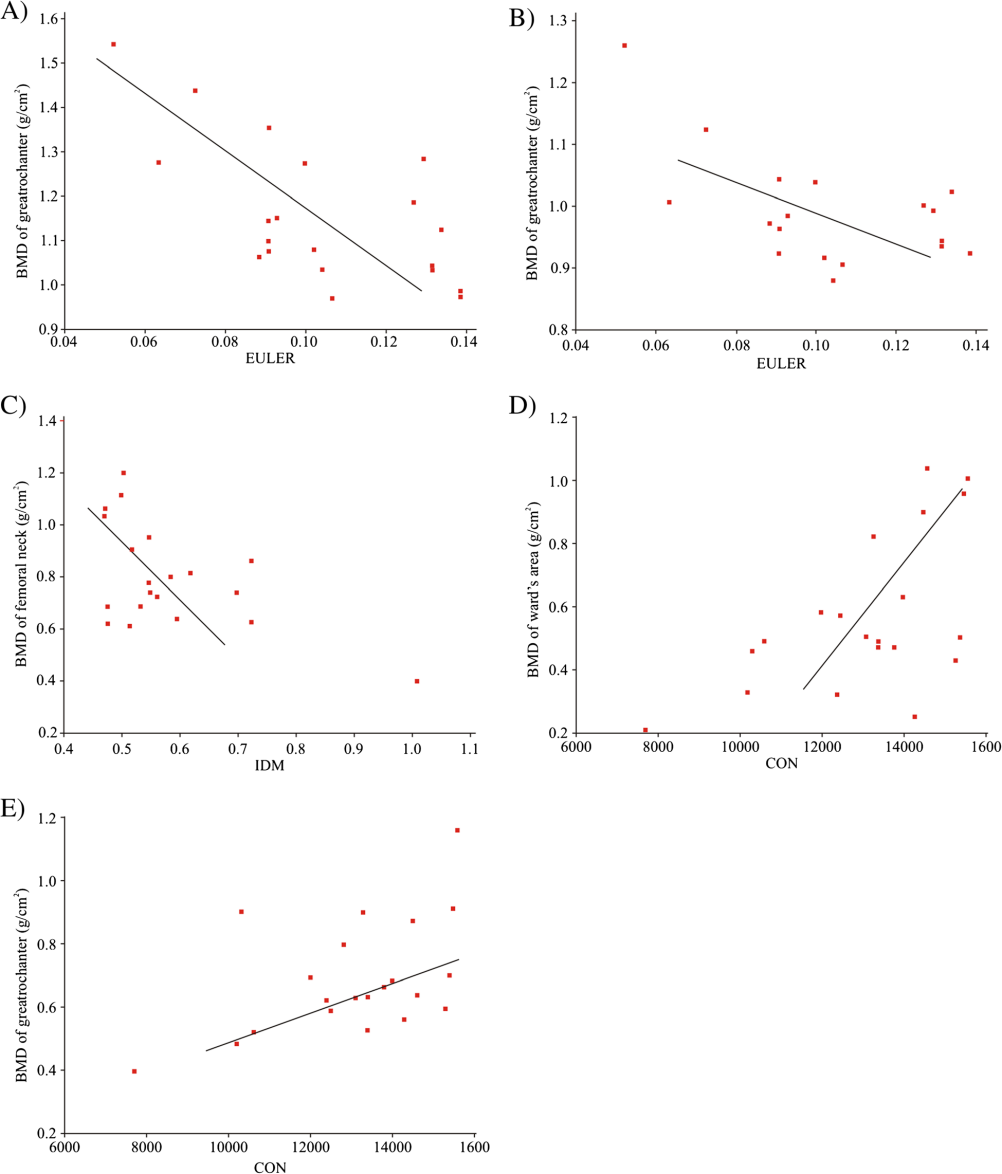
**Relationship between structural and texture parameters and BMD in OA (A and B) and OP patients (C, D and E).** (**A**) BMD of femoral neck with Euler number; (**B**) BMD of Ward’s triangle with Euler number; (**C**) BMD of femoral neck with IDM; (**D**) BMD of Ward’s triangle with contrast; (**E**) BMD of greater trochanter with contrast.

Multiple linear regression analysis showed that in OP group, inverse different moment was able to predict BMD of the femoral neck (r^2^ = 0.305, p < 0.05), while the contrast was also able to predict BMD of the greater trochanter (r^2^ = 0.250, p < 0.05) as well as Ward’s triangle (r^2^ = 0.327, p < 0.01). In OA group, Euler number was the only parameter that was able to predict BMD of either femoral neck or Ward’s triangle (r^2^ = 0.414, p < 0.01; r^2^ = 0.324, p < 0.01) (see Figure 3).

## Discussion

The argument that OP and OA may exhibit an inverse relationship has been proposed 40 years ago. However, the real relationship between these two age-related diseases is still unclear. With the improvement of the understanding of OP, the role of trabecular bone microstructure is widely acknowledged. It is believed that 60% of the mechanical properties of bone depend on bone density, and the remaining may be associated with a number of factors related to bone quality [27,44-47].

In this study, the structural parameters of the two disease groups of women had significant differences in apparent bone volume fraction, trabecular thickness and mean roundness. Our findings also found that trabecular bone number had no statistical significance between OP and OA group. It was reported that trabecular number did not change with age in elderly [48]. Patel *et al.*[49] discovered that knee joint of normal and OA patients does not show any difference in the trabecular number. But, Chappard *et al.*[50] found that late-period OA patient’s trabecular bone number and OP group have significant differences. At the same time, they also believed that it was the loss of OP’s trabecular bone that caused this difference. Stauber and Muller [51] also agreed that the OP patient’s trabecular bone number had dropped. In the ovariectomized animal models, trabecular bone number was usually lower compared to blank comparison group [52].

The thickness of trabecular bone tends to decrease when the trabecular bone space increases. It is generally accepted that trabecular bone thickness reduces along with age, but trabecular bone space gets increasingly wider. At the same time, OP makes this tendency to be increasingly serious regardless of human patients or animal models. Most of the authors think the change in OA patients is just the opposite, namely the trabecular bone thickness increases, which was the same with our results. But interestingly, although the trabecular bone thickness in OA group was higher than in OP group significantly, the difference between their trabecular bone spaces was not statistically significant.

Ding *et al.*[53] found that patients with early OA gained bone volume, while it reduced in normal and OP groups. Although this kind of change may increase the bone volume in OA patients, Dequeker *et al.*[14] thought that low mineralization of the subchondral bone made mechanical characteristics still lower than the normal population. Buckland-Wright *et al.*[54] found that the medial tibial subchondral bone becomes thick in patients with anterior cruciate ligament rupture, while the lateral side had no significant changes. However, in OA patients whether the change of cancellous bone was earlier than that of cartilage was still unclear. Unlike other imaging methods, MRI with multiplanar imaging may intuitively demonstrate all structures within the joints at the same time, and provide help to better understand the relationship between bone and cartilage. Some scholars found that trabecular bone could accurately reflect the degree of bone loss in pigs with arthritis and the trabecular bone underneath the cruciate ligament become thin [55]. Chappard *et al.*[50] found that there was no significant difference in male patients with early OA and OP; on the contrary, there was significant difference between patients with later period OA and OP. They adopted advanced high-resolution Micro-CT system, but patient’s age and gender will possibly influence the comparison result and it could only be studied *in vitro*. It was also found that bone volume fraction is associated with the Young’s modulus [36]. In the current study, we found that apparent trabecular bone volume fraction in OP group was significantly lower than the OA group. Although the mean trabecular bone area in OA is higher than in OP, there was no statistical significance between them.

Roundness is an indicator reflecting the morphology, and roundness of the object is related to value closer to 1. The more this value approaches to 1, the more the structure tends to blunt. As for a network, larger value of roundness implies good connectivity (low perimeter). In the current study, the mean roundness of trabecular bone in women with OP was significantly lower than OA group (p < 0.05), which suggested that the trabecular bone of women with OP was smaller or with less connectivity.

Our research also found that Euler number in the OP group was significantly higher than in OA group, which implied that the connectivity of trabecular bone network structure in OP patients was poorer and bone microstructure was severely damaged. Euler number is the common index reflecting bone connectivity [56]. It may reflect the connection degree of the complex trabecular bone network microstructure. Better the connection of trabecular bone network, lower the value. It could even be negative in a highly connective trabecular bone network [25]. It was also found that the Euler number and other parameters reflecting bone quality and bone structure had remarkable relevance, and they both correlated with the strength of bone [57]. Euler number has nothing to do with bone quantity, but is related with the Young’s module, and may help evaluate cavities and the number of opened or closed loops in bone marrow. They also confirmed that spatial distribution of the cavities and loops in trabecular bone is a major factor of bone strength. In our study, this index was related to mean trabecular bone area in the OP group, but there was no relation with structural parameters in OA group. The multivariate linear regression analysis demonstrated that Euler number was the only parameter, which has linear correlation with BMD in OA group. Contrast and inverse difference moment have linear correlation with BMD in OP group; this suggested that the mechanism of trabecular bone connectivity to bone microstructure and bone quantity under different disease conditions can be very complex and require further study.

We must admit that this research has some limitations. First of all, the sample was still not big enough as a clinical study, and it was insufficient to completely evaluate the real relationship between OP and OA. The heterogeneity of the small sample size is also a drawback. However, we established strict criteria for case selection. Secondly, this series has not been able to integrate ‘completely normal’ control subjects. We also desired to gather age-matched normal people as control group to enhance the effectiveness of comparative studies. But in the same age scope, it was difficult to find postmenopausal women with no disease. The original experimental design intended to determine the erosion index of high-resolution images, which can reflect the degree of destruction of trabecular bone. But, the calculation of erosion index requires special software to carry out the three-dimensional reconstruction of the image. As the software was not currently available domestically, developing software from the beginning costs high and takes a longer time. The significance of Euler number overlaps with erosion index partly. However the index was not included in this study. We hope that with the development of appropriate computer hardware and software, this could be further improved. Lack of data on BMI is also a limitation of the study.

The reason why we chose proximal tibia area to evaluate the trabecular bone microstructure is that this area is more suitable for the coil which is used for HR MRI examination, and the fact that it had been chosen in earlier studies as well. We also tried to evaluate the hip area, but it needs bigger coil, which had poorer quality of HR images. We think it was also feasible to conduct the study had we chosen other area’s BMD, like lumber. However, in our hospital, we examine the hip region as routine.

## Conclusion

In summary, we found significant differences in the microstructure of the trabecular bone between postmenopausal women with OP and OA using HR-MRI at 3 T. These findings not only suggest totally different mechanism and progression of two common diseases, but also support the hypothesis that there is an inverse relationship between OA and OP. Euler number, which reflects the connectivity of trabecular bone network, was found to correlate with quite a few structural or textural parameters. The effect of textural parameters on structure or strength of trabecular bone substantiates the need for further longitudinal studies.

## Competing interests

We declare that there is no competing interest in this study.

## Authors’ contributions

YS carried out acquisition of data, analysis and interpretation of data. YHZ was involved in drafting of manuscript and revision of the same. LS designed and coordinated the study along with drafting the manuscript. All authors read and approved the final manuscript.

## Pre-publication history

The pre-publication history for this paper can be accessed here:

http://www.biomedcentral.com/1471-2474/14/136/prepub

## Supplementary Material

Additional file 1 Texture parameters and image analysis methods. Click here for file
