# Supplementary material for: Postmenopausal women with osteoporosis and osteoarthritis show different microstructural characteristics of trabecular bone in proximal tibia using high-resolution magnetic resonance imaging at 3 tesla
Source: BMC Musculoskelet Disord. 2013 Apr 15;14:136. doi: 10.1186/1471-2474-14-136 (PMC3659090; doi:10.1186/1471-2474-14-136)
Supplement: Additional file 1 — Texture parameters and image analysis methods. [file 1471-2474-14-136-S1.docx]

**Texture Parameters and Image Analysis Methods**

**SGLDM**

Texture was conducted by correlation of neighboring pixel’s gray scale value. Probability of the gray drop related to one pixel can be used to investigate this correlation located in the same geometrical position. For example, as a 2-D digital image with a resolution of M×N, F (i,j) was the gray scale value of the point (i,j). F (i,j) can be demonstrated by the matrix below (Figure 3). For two pixel with d distance, gray scale value as u and v, the probability of appearance in any direction of the image can be expressed as bellows.


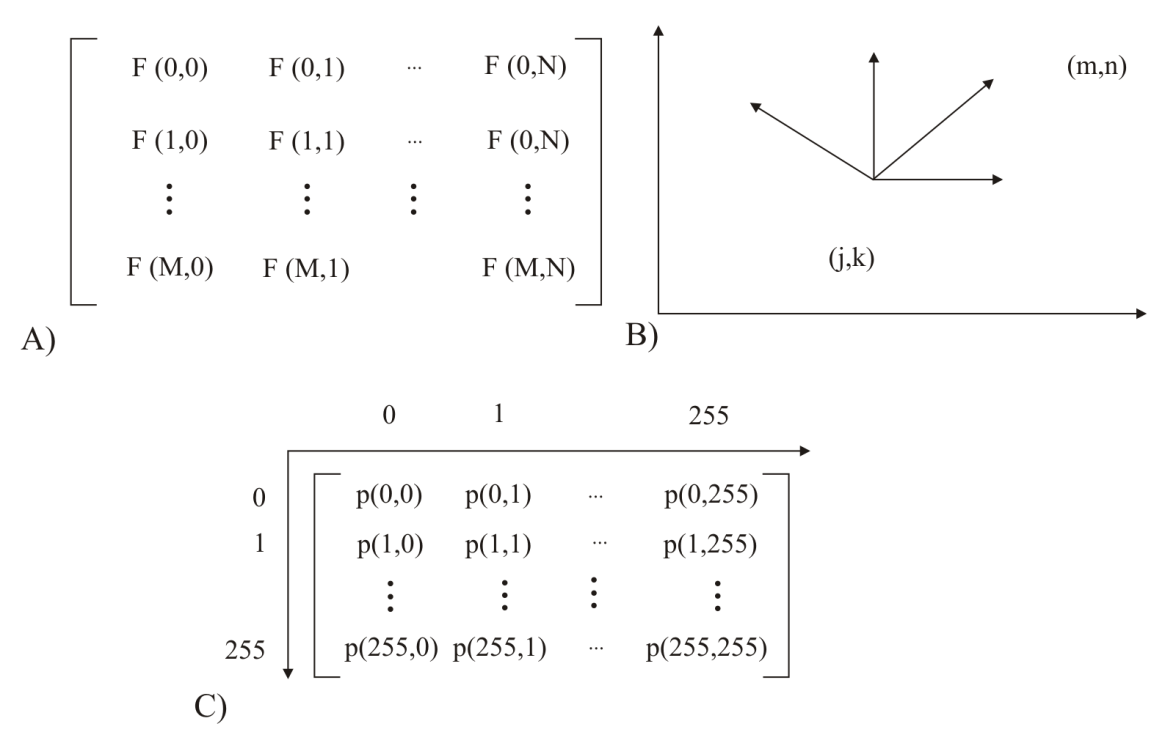


Then we denote parameter d and θ to represent the relative position, where d is the distance of checkerboard distance (later use distance), θ is the angle between the X-axis and the line between two pixels. Suppose 2 pixels X= (j,k) and Y (m,n), like Figure 4, the corresponding grey value is u and v, and the θ for X and Y has 4 cases

1. θ=0, R (d):j-m=-d, k- n=0;

2. θ=45, R (d):j-m=-d, k- n=-d;

3. θ=90, R (d):j-m=0, k-n=-d;

4. θ=135, R (d):j-m=d, k-n=-d;

As for 2 pixels whose grey values are u and v with distance d, the times of it occurs in any direction (which is related with angle θ) can be written as:

P (u, v, d, 0°) =number{ R (d), f (j,k)=u, f (m,n)=v},

P (u, v, d, 45°) =number{ R (d), f (j,k)=u, f (m,n)=v},

P (u ,v, d, 90°) =number{ R (d), f (j,k)=u, f (m,n)=v},

P (u ,v, d, 135°) =number{R (d), f (j,k)=u, f (m,n)=v}

We denote P as Spatial gray level dependence matrix (later denote as S-matrix), which depend on u,v,d, θ. Fix d and θ, since the grey value level is G, u, v would be any integer between 0 to G-1, thus the S-matrix P is a G*G matrix. For instance, SGLDM was a 256×256 matrix in any direction of a 256 gray scale image (Figure 5)

As the setup, we can find out several propositions of the matrix and the meaning for describing the texture information of a picture:

1 S-matrix is a symmetric matrix.

2 S-matrix depends on direction i.e. θ. If θ is different, the corresponding S-matrix is different.

3 The diagonal entry of the S-matrix defined as the times of occurrence of two pixels with same grey values (in fixed direction). The trace of S-matrix is large, means that the change of the grey value in this direction is small, thus the texture is coarse. Otherwise, if the grey values vary greatly, i.e. matrix entry is away from diagonal entry in some sense, the texture is fine.

4. If the values of S-matrix’s entries distribute more at upper-left-corner, or lower-right-corner, i.e. around the diagonal entries, means grey value changes not much, texture is coarse. Conversely, if the values of S-matrix’s entries distribute more at lower-left-corner, or upper-right-corner, i.e. away from diagonal entries, it means, two pixels with large difference between their grey values occur more in this image, the grey values for this image vary greatly and thus texture is fine.

Thus the S-matrix shows the proposition of the texture for a digital image. But the dimension of S-matrix is especially big, depending on the grey value level. Thus, we have to define some variables to describe the property of the S-matrix.

Energy

^^

Energy can describe the uniformity of the texture or grey value of image. If the grey value of the image, or the texture is uniform, the gray value would be lie in a small range, then large value would occur in the S-matrix, thus the energy would be high. Conversely, if not, the S-matrix would have a lot of entries with small values, the energy is relatively low.

Entropy

^^

Entropy represents the chaosity of the image. If the entries of S-matrix are similarly, i.e. the probability for points with all possible grey values are the same in the whole image. Then the texture is, in chaos, thus the entropy is high. Conversely, the texture is regular, the S-matrix only have several large entries. The Entropy is low.

Inertia

*^^*

The Inertia represents the difference of the grey level of the image, like contrast.

Absolute value

*^^*

Absolute value describes the complexity of the image. If it’s higher, the image is more complicated.

Inverse difference

*^^*

Inverse difference describes the aggregation density of an image.

Difference of image size or grey value level would lead to huge difference in every entries, thus we have to normalize the entries, before we use these variables. We say, Sum-1-condition.

*^^*

*^^*

**Grey level difference matrix (G-matrix)**

Grey level matrix summarized the absolutely difference between grey values of two adjacent Pixels. It also can be describe the property of texture value.

The way to compute it is following. Suppose a digital image f (i,j), given (*∆i, ∆j*), let *f´* (*i,j*) = |*f* (*i,j*)-*f* (*i+∆i*,*j+∆j*)|. Summarize all values of *f´* (*i,j*), and get the relative probability, then get the G-matrix p (i). For example, if the image has grey value level G, the G-matrix has size 1*G. and the value of component n is just the probability of *f´* (*i,j*)=n

The meaning of G-matrix is very clear, if the texture of an image is clear, then the grey value changes frequently, then the distribution of the values in matrix would be centered mostly at where *i* is large or be very separately. Conversely, for coarse image, the distribution would be centered at where *i* is small.

Here are the variables based on G-matrix

Contrast

*^^*

If the contrast of the image grey value is large, then the difference of gray value for adjacent pixels is large, then the distribution of large value of G-matrix would be centered at where *i* is large, then the contrast is large, conversely, the contrast is small.

Angular Second Moment

*^^*

This parameter has similar meaning as energy.

Inverse Different Moment

*^^*

This parameter describes the uniformity in certain area.

**Figure Legends:**

Fig. 3A: Gray-scale value (F) matrix.

Fig. 3B: Relative position d and θ.

Fig. 3C: Spatial gray-level dependence matrix (P).
